# Homing Pigeons Respond to Time-Compensated Solar Cues Even in Sight of the Loft

**DOI:** 10.1371/journal.pone.0063130

**Published:** 2013-05-24

**Authors:** Chris Armstrong, Helen Wilkinson, Jessica Meade, Dora Biro, Robin Freeman, Tim Guilford

**Affiliations:** 1 Department of Zoology, University of Oxford, Oxford, United Kingdom; 2 School of Biological and Biomedical Sciences, Durham University, Durham, United Kingdom; 3 School of Biological, Earth and Environmental Sciences, University of New South Wales, Sydney, Australia; 4 CoMPLEX, UCL Gower Street, London, United Kingdom; Bowling Green State Universtiy, United States of America

## Abstract

The sun has long been thought to guide bird navigation as the second step in a two-stage process, in which determining position using a map is followed by course setting using a compass, both over unfamiliar and familiar terrain. The animal’s endogenous clock time-compensates the solar compass for the sun’s apparent movement throughout the day, and this allows predictable deflections in orientation to test for the compass’ influence using clock-shift manipulations. To examine the influence of the solar compass during a highly familiar navigational task, 24 clock-shifted homing pigeons were precision-tracked from a release site close to and in sight of their final goal, the colony loft. The resulting trajectories displayed significant partial deflection from the loft direction as predicted by either fast or slow clock-shift treatments. The partial deflection was also found to be stable along the entire trajectory indicating regular updating of orientation via input from the solar compass throughout the final approach flight to the loft. Our results demonstrate that time-compensated solar cues are deeply embedded in the way birds orient during homing flight, are accessed throughout the journey and on a remarkably fine-grained scale, and may be combined effectively simultaneously with direct guidance from familiar landmarks, even when birds are flying towards a directly visible goal.

## Introduction

Biological compasses allow animals to use external reference cues to “select and maintain specific directions” [1] during movement. Clock-shifting experiments have been used to demonstrate that a time-compensated sun-compass is widespread in animals [2]–[4], particularly the homing pigeon model where predictable deflections in initial orientation are reported over unfamiliar [5] and familiar terrain [6] in anosmic [7], naïve [8] and hippocampal-lesioned subjects [9], and in magnetically disturbed birds [10]. Broadly, the evidence shows that under sunny skies birds respond to the sun’s azimuthal direction, but not its height, and can compensate fully for the sun’s apparent movement across the sky during the day by referencing their internal circadian clock (a process that requires previous experience of sun’s movement pattern [11]). However, partial deflection has been observed in numerous experiments (reviewed in [12]), and is normally thought to be caused by orientation that is a compromise with conflicting cues either from learnt arrangements of landmarks, or from an alternative unshifted (magnetic) compass [13]. Compass orientation is particularly valuable for long-distance movements (such as migration, or navigation from unfamiliar places), because it allows the maintenance of goal-ward progress without the need to update position constantly. Naïve migrants may use innately instructed compasses to reach very distant over-wintering sites [14]–[17], whilst for true navigation over unfamiliar terrain, Kramer’s [18] two-stage map-and-compass concept has remained the dominant explanatory model [19], [20]. Over short distances, and particularly over familiar terrain, the functional utility of compass orientation is less clear since landmarks can provide an independent source of directional guidance. Nevertheless, the two-step model is still generally accepted as the explanation for deflected orientation because animals may use familiar landmarks to fix position and a compass to maintain orientation between known locations, a process referred to as the “mosaic map” [21]. To investigate the function of solar guidance in avian homing more critically we clock-shifted pigeons under conditions in which compass orientation might be predicted to have no value: over familiar terrain close to and in direct sight of the final goal.

## Results

**Figure 1:**
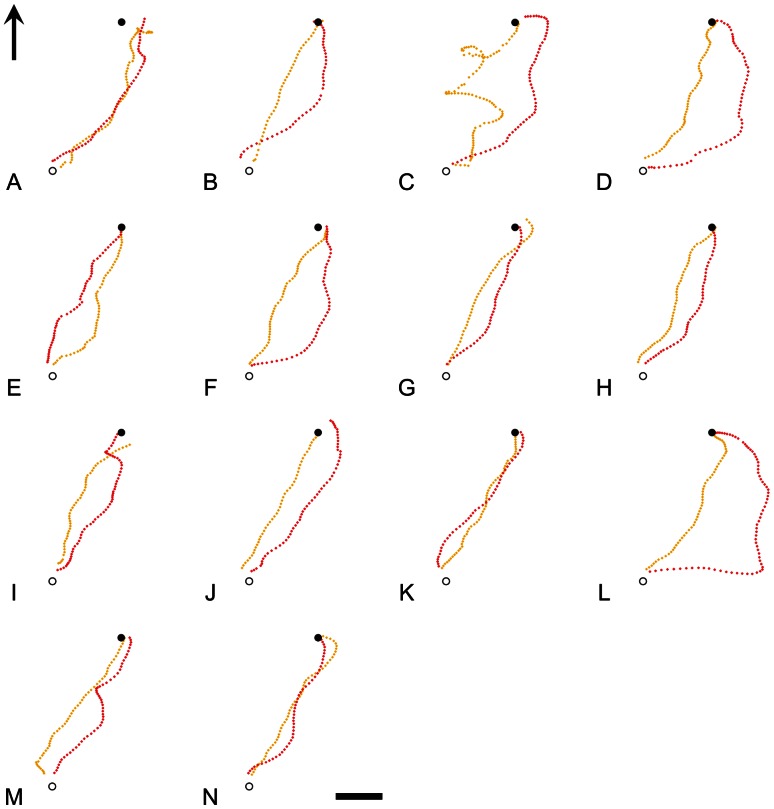
Individual slow clock-shifted trajectories show partial deflection in the predicted direction. GPS-derived tracks of individual pigeons overlaid on satellite images of the area surrounding the University of Oxford Field Station, Oxfordshire, UK, including the release site (open circle) and the loft (filled circle). Panels show individual subjects’ control (yellow tracks) and first slow clock-shift release for the slow-shifted group (red tracks). The arrow in the first panel signifies North and the scale bar in the final panel represents 200 m.

**Figure 2:**
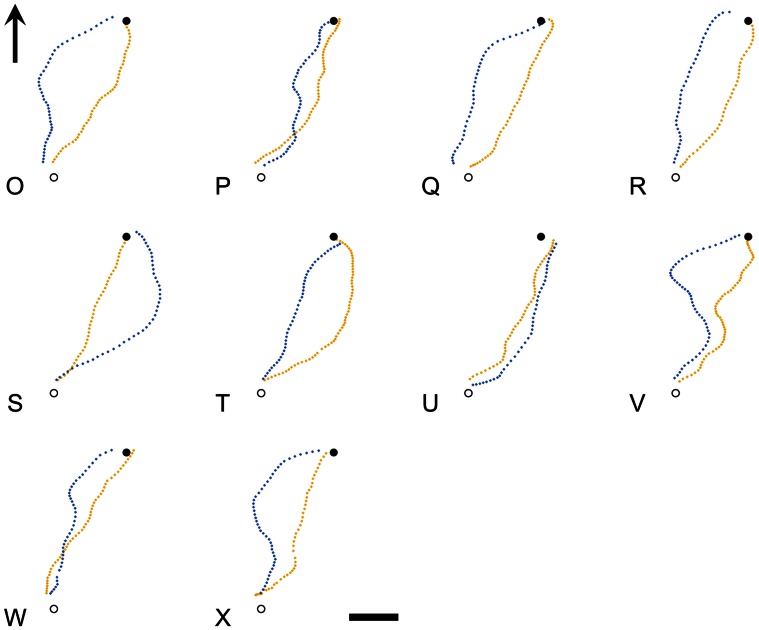
Individual fast clock-shifted trajectories show partial deflection in the predicted direction. GPS-derived tracks of individual pigeons overlaid on satellite images of the area surrounding the University of Oxford Field Station, Oxfordshire, UK, including the release site (open circle) and the loft (filled circle). Panels show individual subjects’ control (yellow tracks) and first fast clock-shift release for the fast-shifted group (blue tracks). The arrow in the first panel signifies North and the scale bar in the final panel represents 200 m.

**Figure 3:**
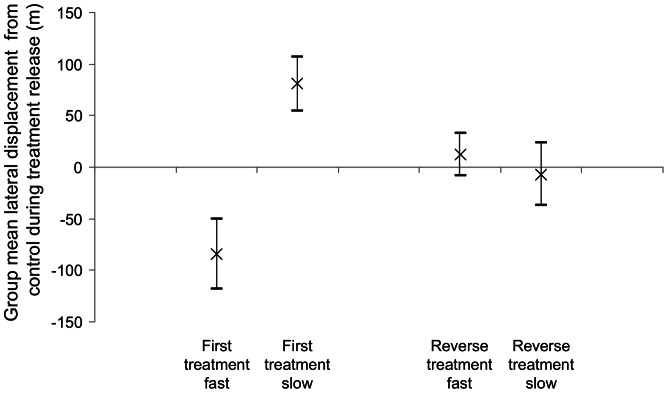
The clock-shift effect is not observed after a reverse shift treatment. The treatment group means of the individual subjects’ mean lateral trajectory displacement from control are displayed for each treatment group. Lateral displacement from control is calculated using a nearest neighbour analysis, where neighbour distances deflected in the anti-clockwise direction from control are assigned negative values. The error bars represent the standard error.

On their first clock-shifted release, the birds, as a group, were consistently partially deflected from their un-shifted trajectories (yellow trajectories in Figures 1 and 2) in the direction predicted by the time-compensated compass: anti-clockwise for slow-shifted birds (red trajectories in Figure 1) and clockwise for fast-shifted birds (blue trajectories in Figure 2). There is variation amongst subjects in the degree (and even direction) of difference between control and treatment trajectories, and whilst an interesting possibility is that this is due to individual differences in response to clock-shift treatments, it cannot easily be distinguished from the effects of noise since the design precluded repeated measures on the same individuals. We compared each bird’s trajectory under clock-shift to its own control trajectory by calculating the distance between every point on the shifted trajectory and its nearest neighbour on the control trajectory. Offset distances in the anti-clockwise direction from control were arbitrarily labelled negative, to distinguish deflections either side of the control, such that a trajectory with equally sized sections either side of the control trajectory would generate an average offset distance of zero. Mean offset was significantly in the clockwise direction for slow-shifted birds (group mean  = 81 m, p  = 0.001 for a 2-tailed Z-test: Zstat  = 3.08, for a Zcrit of 1.96 with 13 df), significantly in the anti-clockwise direction for fast-shifted birds (group mean = −85 m, p  = 0.006 for a 2-tailed Z-test: Zstat = −2.49, for a Zcrit of −1.96 with 9 df) and significantly different between the two treatments (p<0.001 for a 2-tailed T-test: Tstat  = 3.85, for a Tcrit of 2.10 with 19 df. See Figure 3). The broadly symmetrical result here between fast and slow-shifted birds demonstrates that this deflection cannot be a general response to the clock-shift treatment, or a site-specific bias, but instead must be caused by a false reading of solar-based directional guidance information. Thus, even though the birds are familiar with the general area around their lofts, have been released once already from the site (control condition), and have a direct line of sight to the final goal- all conditions which should promote the use of memorised landscape information- time-compensated solar information is still involved in guiding orientation. Subsequent reverse-shifting of each bird appears to abolish this effect (Figure 3).

## Discussion

Smaller-than-predicted deflection under clock-shift is commonly seen in pigeons flying from familiar sites [7], [12], [22]–[25]. The continued deflection is usually explained in terms of the continued use of a two-step map-and-compass system in the form of a “mosaic map” of landmarks (most likely mainly visual) used to assess position followed by the use of a sun compass to guide orientation to the next or final goal [19]. The reduced deflection has sometimes been attributed to a compromise between the birds’ solar and magnetic compasses [13], [26], or, especially in the very familiar area, (and where access to the olfactory map has been abolished through anosmia) to a compromise with learned compass-independent spatial guidance by landmarks (“pilotage”) [7], [24], [27]. Differences amongst individuals in response to clock-shifting, suggesting a mix of individuals using alternative orienting strategies (e.g. [25]), could also contribute to a reduced group mean deflection. In fact, very few studies have claimed an absence of deflection under clock-shift. It was suggested for a familiar site 9.3 km from the loft [25], but the accuracy of the early tracking devices used (heading recorders), and the small and between-subject design may have obscured the kinds of small effects shown here and in other studies [27]. The only situation in which a lack of clock-shift deflection is generally agreed concerns Graue’s [28] previous attempt to release clock-shifted birds in direct sight of the loft. Keeton [29] also reports results which he concludes to be consistent with Graue’s finding, but direct line of sight to the loft was only conjectured. We believe these results may also be an artefact of the inadequacies of the traditional vanishing bearing method of gauging orientation. Measuring vanishing bearings is particularly problematic over such short distances, since in sight of the loft it is seemingly inevitable that birds will have corrected anything but a major deflection before disappearing from sight. Our results at once confirm the fact that attention to the sun compass is reduced where familiar landmark cues are available, yet at the same time strengthen the view that time-compensated solar information is deeply embedded in the way birds orient, even if it only controls orientation partially, seemingly removing the one outstanding exception to date.

**Figure 4:**
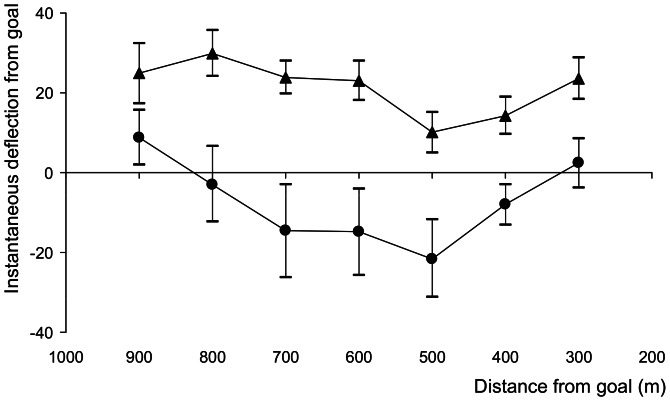
Deflection from homeward is maintained throughout the homing flight. Clock-shifted group means of the instantaneous deflection from home are displayed as a function of distance from the loft. Deflection was defined as the difference between the instantaneous flight heading (measured over a two second rolling average) and the bearing of the loft from that position. Mean deflection values for each subject were averaged within 100 m circular bins, measured from the loft, for the fast-shifted group (circles) and the slow-shifted group (triangles). The error bars represent the standard error.

Our experiment allows a closer examination of the role of solar guidance in the familiar area. Because we released birds very close to the final goal (home), the instantaneous homeward bearing changes relatively rapidly on any straight trajectory as it progresses away from release in all realistic cases except perfectly homeward initial orientation. Birds that have set a deflected compass course after initial position fixing at release should show an increasing instantaneous deviation from the homeward direction as they progress, until they eventually correct (which they must do to arrive at home). Crucially, this is true even if the initial compass course is only partially responsible for the actual trajectory, perhaps because it is combined with direct guidance from familiar visual landmarks. On the contrary, we found that instantaneous relative deviation from the homeward direction in fact remains remarkably constant throughout the homing flight (Figure 4), indicating that whatever forces the initial deflection, continues to update its influence throughout the flight. Remarkably, this remains true even in the final 300 metres, when the home target is so large and close that we reach the limit of our ability to measure confidently the assumed homeward bearing. Put simply, clock-shifted birds do not follow a compass influenced direction away from release towards home ending in a final correction. Instead, birds follow a curving trajectory, more or less constantly readjusted as the effective deviation from home of the current heading increases with proximity to the loft.

In terms of a two step map-and-compass process, such results might be achieved by a series of closely spaced position fixes followed by equally frequent compass course re-assessments (which remain deflected). With each re-assessment of position and course, the bird perceives a new discrepancy between its current course and goalward, and adjusts. Because of the clock shift however, each new compass instruction is itself deflected and, when compromised with the conflicting guidance from the arrangement of landmarks themselves, results in a new course which is deflected slightly from homeward. If so, this implies that the familiar area map-and-compass system can be remarkably fine-grained. Strictly, such a process remains concordant with partial control under the “mosaic map” model [21] in terms of the two navigational inputs (familiar landmarks provide position fixes, whilst the sun-compass provides at least partial directional guidance). However the spatiotemporal scale over which these inputs must be consulted successively to account for the observed results is remarkably fine. In previous work we showed that the time-compensated solar compass helps control orientational guidance decisions when birds pilot along thoroughly familiar routes home at a scale of minutes and a few kilometres [27]. Here we have shown that this continues to be the case even on a scale of a few seconds and metres, and when the goal itself is directly in sight. Whilst the precise mechanisms by which birds combine time-compensated solar cues and direct landmarks remain to be resolved, it is clear from our results that these two sources of guidance information can operate on similarly fine temporal and spatial scales, and perhaps even simultaneously.

A secondary result from our experiment is that the deviation under clock-shift appears to be abolished, or at least reduced below our ability to detect it, when birds are subsequently reverse shifted and released again. This could indicate that birds reduce further their dependence on solar cues as they become yet more familiar with the landmark arrangements during this short homing task, or because they have increased experience of their sun-compass providing faulty directional information, but these alternatives are confounded here and will need to be tested directly using a different experimental design.

## Materials and Methods

### Ethics Statement

The experimental protocol of attaching telemetry devices to the backs of pigeons at our loft for experiments such as this one was approved by Oxford University's Local Ethical Review Committee (Zoology) at its 17th May 2007 meeting (approval number OULERC-ZOO-17052007). Approval for specific experiments outside A(SP)A are not required either by law or by the official ethical review process. This experiment was conducted in accordance with the approved ethical review process.

### Subjects and Materials

A mixed age (minimum 2 years and 480 g) group of 30 homing pigeons were selected at random from the colony at the University of Oxford Field Station at Wytham (51°46′58.34″N, 1°19′02.49″W). All subjects were experienced with the loft area and surrounding countryside, and had participated in previous homing studies, although none had been released from the experimental release site. Established methods were employed to prepare the subjects for carrying GPS loggers [30], and immediately prior to the experiment the subjects were given four individual training flights from sites <3 km from the loft in widely dispersed directions and carrying training weights.

### Release Site

The subjects were equipped with on-board miniature GPS loggers, weighing 24 g and set to record time-stamped position fixes at 1 Hz. The subjects were then released under normal and 6-hour clock-shifted diurnal cycles, from a release site (51°46′29.37″N, 1°19′18.76″W) within direct sight of their loft (950 m away, bearing to loft 26.2°). Releases took place while the sun’s disc was visible and during the period of overlap between the subject’s real and perceived daytimes. In the case of logger failure, the trajectory was eliminated from the analysis. The GPS track data are available through the Dryad repository (http://dx.doi.org/10.5061/dryad.pc7pg).

### Release Treatments

The subjects were randomly allocated to two groups of 14 receiving different treatment orders. Subjects received 1 control release under a normal diurnal cycle, followed by a release under either slow shift (6 hours behind) or fast shift (6 hours ahead), and followed again by a release in which the clock-shift treatment was reversed. All releases were separated by six days free-flight and six days of confinement to the clock-shift lofts.
